# Noninvasive Monitoring of Bleomycin-induced Lung Injury in Rats Using Pulmonary Function Test

**DOI:** 10.5487/TR.2008.24.4.273

**Published:** 2008-12-01

**Authors:** Mi-Jin Yang, Young-Su Yang, Yong-Bum Kim, Kyu-Hyuk Cho, Jeong-Doo Heo, Kyuhong Lee, Chang-Woo Song

**Affiliations:** Division of Inhalation Toxicology, KIT Jeongeup Campus, Jeongeup, Jeollabuk-do, 580-185 Korea

**Keywords:** Pulmonary function, Respiratory frequency, Tidal volume, Minute volume, Bleomycin, Animal model, Noninvasive monitoring, Lung injury

## Abstract

The single intratracheal instillation (ITI) of bleomycin (BLM) is a widely used method for inducing experimental pulmonary fibrosis in rat model. In the present study, pulmonary function tests (PFTs) of tidal volume (V_T_), minute volume (V_M_), and respiratory frequency (F_R_) have been applied to study their possibility as a tool to monitor the progress of BLM-induced lung injury in rat model. Rats were treated with a single ITI of BLM (2.5 mg/kg) or saline (control). Animals were euthanized at 3, 7, 14, 21, and 28 days post-ITI. Lung toxicity effects were evaluated by inflammatory cell count, lactate dehydrogenase (LDH) activity in the bronchoalveolar lavage fluid (BALF), and light microscopic examination of lung injury. The PFT parameters were measured immediately before the animals were sacrificed. BLM treatment induced significant cellular changes in BALF-increase in number of total cells, neutrophils, and lymphocytes along with sustained increase in number of macrophages compared to the controls at days 3, 7, and 14. BALF LDH level was significantly increased compared to that in the controls up to day 14. On day 3, infiltration of neutrophils was observed in the alveolar spaces. These changes developed into marked peribronchiolar and interstitial infiltration by inflammatory cells, and extensive thickening of the interalveolar septa on day 7. At 14, 21, and 28 days, mild peribronchiolar fibrosis was observed along with inflammatory cell infiltration. The results of PFT show significant consistencies compared to the results of other toxicity tests. These data demonstrate that the most suitable time point for assessing lung fibrosis in this model is 14 days post-ITI of BLM based on the observation of fibrosis at 14, 21, and 28 days. Further, the progress of lung injury can be traced by monitoring the PFT parameters of F_R_, V_T_, and V_M_.

## INTRODUCTION

Idiopathic pulmonary fibrosis (IPF) is characterized by lung inflammation and abnormal tissue repair brought about by cytokines and interactions between several cell types, resulting in the expression of collagen gene and subsequent collagen deposition in the lungs (Piguet *et al.*, 1990; Gross and Hunninghake, 2001; Gharaee-Kermani and Phan, 2005). Although the pathogenesis of IPF is related to immune cells, extracellular matrix repair, cytokines, chemokines, etc, the exact mechanism of the disease remains unclear (Zhang *et al.*, 1994a, b, 1996; Kuwano *et al.*, 2001; Gharaee-Kermani and Phan, 2005; Gharaee-Kermani *et al.*, 2008).

IPF is an irreversible disease with poor survival rates, and therapeutic methods such as use of anti-inflammatory agents (Mason *et al.*, 1999; Lynch *et al.*, 2001), anti-fibrotic agents (Okada *et al.*, 1993; Antoniou *et al.*, 2003), receptor antagonist (Kolb *et al.*, 2001), signal transduction inhibitors (Nakao *et al.*, 1999), etc are not very effective. Further, the pathogenesis of IPF and efficient therapeutic agents against IPF have not yet been determined.

Bleomycin (BLM) is a potent chemotherapeutic antitumor agent derived from *Streptomyces verticillus* (Adamson, 1976; Umezawa, 1967); however, it is known to induce toxic side effects such as pulmonary fibrosis in humans as well as in experimental animals. Therefore, the BLM-induced pulmonary fibrosis model is widely used for the study of the mechanism of the disease and its treatment (Jordana *et al.*, 1988; Sakanashi *et al.*, 1994; Gharaee-Kermani *et al.*, 2005; Moeller *et al.*, 2008). In animal models, it is very important to determine the onset and severity of inflammation and fibrosis in studies of pathogenesis and therapy since the severity of induced disease using the same method and dose of BLM can be different from the genetic specificity within a species, the experimental environments, and the skillfulness of agent instillation. Furthermore, a non-invasive monitoring method is needed for the selection of proper animals with adequate severity of disease.

Magnetic resonance imaging (MRI) (Karmouty-Quintana *et al.*, 2007) and computed tomography (CT) (El Maghraoui *et al.*, 2004) are non-invasive techniques used to monitor the progress of fibrosis. Inflammation and morphological changes, in particular, have been effectively monitored using the change of lung parenchymal signals, indicating that MRI is a good tool to evaluate the therapeutic effects in experimental lung fibrosis rat model. Pulmonary function test (PFT) (Lopez-Majano and Renzi, 1978; Flaherty and Martinez, 2000; Martinez and Flaherty, 2006; Nathan *et al.*, 2007) is an alternative way to non-invasively monitor the physiological state of animals, and is very sensitive to inflammation in lungs (Seagrave *et al.*, 2008) and temporal influx of immune cells into lungs (Spond *et al.*, 2004).

The aim of the present study is to establish a guideline for single ITI BLM-induced lung fibrosis model in Sprague-Dawley (SD) rats. Lung damage was assessed by several techniques, including total cell count, differential count of inflammatory cells in BALF, and measurement of LDH activities and histopathological examination. In addition, PFT of respiratory frequency ($F_R$), tidal volume ($V_T$), and minute volume ($V_M$) were introduced to non-invasively monitor the progress of BLM-induced lung fibrosis in animal models.

## MATERIALS AND METHODS

***Animals.*** SD male rats (n = 50, Orient bio, Sungnam, Korea), 6 weeks old, weighing 190 to 220 g, were used in this study. All the animals were kept on a diet of Lab 5053 (Orient bio, Sungnam, Korea) and filtered tap water. Rats were housed in a HEPA-filtered clean air, viral- and antigen-free room and kept in a temperature-controlled environment (23°C ± 3°C) with an alternating 12-h light/dark cycle. Rats were acclimated to these conditions for 1 week before ITI of BLM. All the animal facilities in this study were accredited by the Association for Assessment and Accreditation of Laboratory Animal Care International (AAALAC).

***Experimental design.*** Bleomycin sulfate (Sigma-Aldrich, St. Louis, USA, lot# B2434) was dissolved in sterilized 0.9% saline and administered intratracheally as a single dose of 2.5 mg/kg in 0.1 ml solution per animal. The dose of BLM was selected from previous experiments conducted in this laboratory so as to cause no mortality but consistent biochemical and histological damage (Cortijo *et al.*, 2001). Control animals received 0.1 ml saline only. Intratracheal instillation was carried out under isoflurane anesthesia.

Rats were randomly assigned to five weight-matched experimental groups-5 groups for 3, 7, 14, 21, and 28 days post ITI-BLM (n = 5) and 5 more groups for ITI-saline as a control. On 3, 7, 14, 21, and 28 days following ITI BLM or saline instillation, animals were sacrificed by exsanguinations of the abdominal aorta under an overdose of isoflurane. Lung injury was evaluated by analysis of bronchoalveolar lavage fluid (BALF) and light microscopic examination (BX51, Olympus, Tokyo, Japan) as follows.

***Bronchoalveolar lavage (BAL).*** BAL was carried out on rats from each group as previously described (Antonini *et al.*, 2004; Yang *et al.*, 2008). The rats were euthanized with isoflurane and exsanguinated by severing the abdominal aorta. The left lung was clamped off and the right lungs were washed out twice with 3 ml of warm, calcium- and magnesium-free phosphate buffer solution (PBS, pH 7.4; Sigma-Aldrich, St. Louis, USA). The BALF samples obtained were centrifuged (500 g, 10 min, 4°C). The LDH level of the resultant cell-free supernatant was analyzed as described below. The right lungs were lavaged 4 more times with 3 ml of PBS and these samples were centrifuged (500 g, 10 min, 4°C) to obtain the cell pellet only. The cell pellets from all recovered lavage fluids for each rat were combined and resuspended in 1 ml of PBS in order to evaluate the cellular parameters.

***Cellular evaluation of BAL cells.*** The cells recovered by BAL were counted and identified. The total BAL cell numbers were measured using a cell viability analyzer (Berkman Coulter, Miami, FL). BAL cells were spun at 800 rpm for 5 min and pelleted onto a slide using a cytospin 4 centrifuge (Thermo-Shandon, Pittsburgh, PA). Cells (300/rat) were differentially counted as alveolar macrophages (AM), polymorphonuclear neutrophils (PMN), and lymphocytes after staining with Wright-Giemsa Sure stain (Muto Pure Chemicals, Tokyo, Japan).

***Analyses of LDH level.*** The activity of the cytosolic enzyme, lactate dehydrogenase (LDH), as indicator of pulmonary damage within the first cellular supernatant fraction of BALF, was assayed to detect general cytotoxicity. LDH measurements were performed using DRY-CHEM 3500S (Fuji Film, Tokyo, Japan). The measurements were performed according to the manufacturer's instructions.

***Histopathologic evaluation.*** After collection of BALF, the right lungs were perfused intratracheally with 10% neutral phosphate buffered formalin (NPBF; Formalin, Yakuri Pure Chemicals, Kyoto, Japan; Sodium phosphate dibasic, Samchun Pure Chemical, Pyeongtaek, Korea; Sodium phosphate monobasic, Pyeongtaek, Korea) and fixed in 10% NPBF pending process. Specimens were dehydrated and embedded in paraffin, sectioned into 3 μm-thick slices onto microscope slides, and stained with hematoxylin and eosin (H&E). Collagen deposition was assessed using Masson's Trichrome staining (Sigma-Aldrich, St. Louis, Mo) as previously described.

Histologic grading of lesions was performed by two experienced histopathologists using a blinded semi-quantitative scoring system for extent and severity of inflammation and fibrosis in lung parenchyma, as previously outlined (Szapiel *et al.*, 1979; Ashcroft *et al.*, 1988). Briefly, four lung sections from each animal were scored using the following grading scheme. Criteria for grading pulmonary fibrosis were as follows: grade 0 for normal tissue, grades 1~5 for presence of pulmonary inflammation and fibrosis. The severity of lesions was graded as 1 (minimal), 2 (mild), 3 (moderate), 4 (marked), and 5 (severe), and the extent of inflammatory lesions was graded as 1 (10% of the slide), 2 (10%~20%), 3 (20%~40%), 4 (40%~70%), and 5 (70% of tissue affected).

Criteria for grading pulmonary fibrosis were as follows: Grade 0 = normal lung; Grade 1 = minimal fibrous thickening of alveolar or bronchiolar walls; Grade 2 = mild fibrous thickening of alveolar or bronchiolar walls without obvious damage to the lung architecture; Grade 3 = increased fibrosis with definite damage to the lung structure and the formation of fibrous bands or small fibrous masses; Grade 4 = severe distortion of the lung structure and large fibrous areas; and Grade 5 = total fibrous obliteration of the field.

***Lung function test.*** For the study of the mechanical properties of the respiratory system *in vivo*, a small animal whole body pressure plethysmograph (Buxco Electronics, North Carolina, NY) was used. Pressure changes in the plethysmograph were detected using a transducer. Changes in lung volume were calculated depending on the pressure changes in the box. Airflow was calculated by electronic derivation. All variables were collected using BioSystem XA software (Buxco Electronics, North Carolina, NY) at 30 samples per second. From the time course trace of volume change, the respiratory frequency ($F_R$) was analyzed, and tidal volume ($V_T$), minute volume ($V_M$) were calculated.

***Statistical analysis.*** Values were expressed as means ± SD. For analysis of differences between the groups, ANOVA followed by Student's *t* test for individual comparisons between the groups was performed. Statistical significance was established when $p < 0.05$ and $p < 0.01$.

## RESULTS

***Body weight changes.*** All the animals had survived for the whole study duration of 28 days. Rats showed a significant decrease in their body weight during the first 3 days after ITI-BLM and sustained less body weight than the saline-treated control group with significant ($p < 0.01$ or $p < 0.05$) difference (data not shown). No distinct abnormal behavior after treatment was observed.

***BALF analysis.*** Total and differential BALF cell counts of each day are presented in Table 1. Recovery rates of BALF ranged from 95% to 100% and were not

**Table 1.** Total and differential cell counts and LDH value in BALF for the each group of animals at 3, 7, 14, 21, and 28 days

| Parameters \ Day | Day 3 | | Day 7 | | Day 14 | | Day 21 | | Day 28 | |
|---|---|---|---|---|---|---|---|---|---|---|
| | CON | BLM | CON | BLM | CON | BLM | CON | BLM | CON | BLM |
| Total cells (x $10^4$/ml) | 6.4 (3.3) | 39.8 (7.9)** | 5.0 (2.4) | 56.4 (8.1)** | 9.2 (2.7) | 27.6 (22.2) | 12.2 (3.56) | 17.0 (8.8) | 8.2 (1.3) | 14.3 (12.2) |
| Macrophages | 6.0 (3.1) | 17.9 (3.5)** | 4.6 (2.2) | 23.6 (3.4)** | 8.6 (2.6) | 20.7 (16.6) | 11.4 (3.3) | 14.1 (7.3) | 7.6 (1.2) | 12.3 (10.4) |
| Neutrophils | 0.1 (0.1) | 13.1 (0.6)** | 0.2 (0.1) | 25.3 (3.6)** | 0.3 (0.1) | 4.7 (3.7) | 0.4 (0.1) | 1.7 (0.8) | 0.2 (0.0) | 1.2 (0.1) |
| Lymphocytes | 0.1 (0.1) | 3.2 (0.6) | 0.1 (0.1)** | 6.8 (1.0)** | 0.3 (0.1) | 2.0 (1.6) | 0.3 (0.1) | 1.2 (0.6) | 0.3 (0.0) | 0.9 (0.7) |
| LDH (U/l) | 14.4 (2.1) | 46.8 (8.5)** | 16.6 (2.6) | 72.8 (23.5)** | 14.2 (4.1) | 26.8 (10.0)* | 20.2 (2.94) | 19.3 (4.9) | 17.0 (2.9) | 20.6 (8.1) |

Data presented as mean ± S.D. of groups, CON = control, instilled with saline, BLM = treatment, instilled with BLM.
*$p < 0.05$ vs. saline control group.
**$p < 0.01$ vs. saline control group.

significantly different between the BLM-ITI and control groups. The difference in total cell number, alveolar macrophages, neutrophils, and lymphocytes compared to controls was increased in BLM-instilled rats at day 3 ($p < 0.01$), was maximum at day 7 ($p < 0.01$), decreased at day 14 and was minimum at days 21 and 28, as shown in Fig. 1(A). On days 3 and 7, neutrophils and alveolar macrophages were the major cellular components. However, on day 14, alveolar macrophages were the major cellular components, whereas neutrophils were the lowest in number. There was similar change in the number of lymphocytes but the absolute number was small compared with that of total cell.

The LDH activity in BALF, which represents the extent of lung cell damage, was significantly ($p < 0.05$ or $p < 0.01$) increased in rats following ITI-BLM for 14 days compared with control (Table 1). On day 7, rats instilled with BLM showed the highest LDH content compared to groups on other days, as shown in Fig. 1(B).

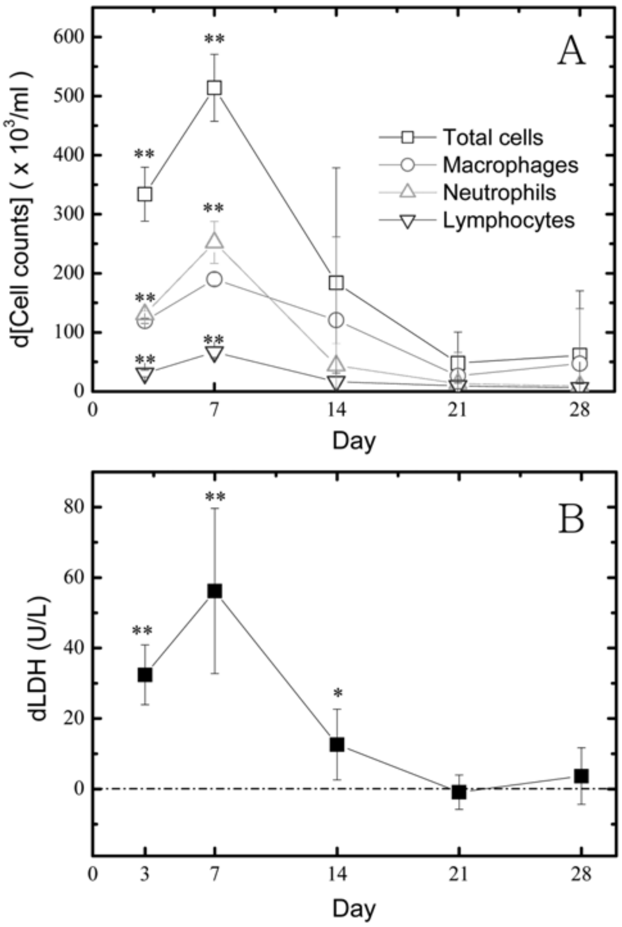

**Fig. 1.** BALF cell distribution and LDH activity in rats over 28 days post-instilled bleomycin, (A) total and differential cell counts (alveolar macrophages, neutrophils, lympocytes), (B) LDH level in BAL fluid. The dash-dot line in (B) for the guide to the eye.

***Lung function test.*** The difference in respiratory function data between the control and BLM-instilled groups is shown in Fig. 2. The difference in respiratory frequency ($dF_R$) was significantly ($p < 0.01$) increased at

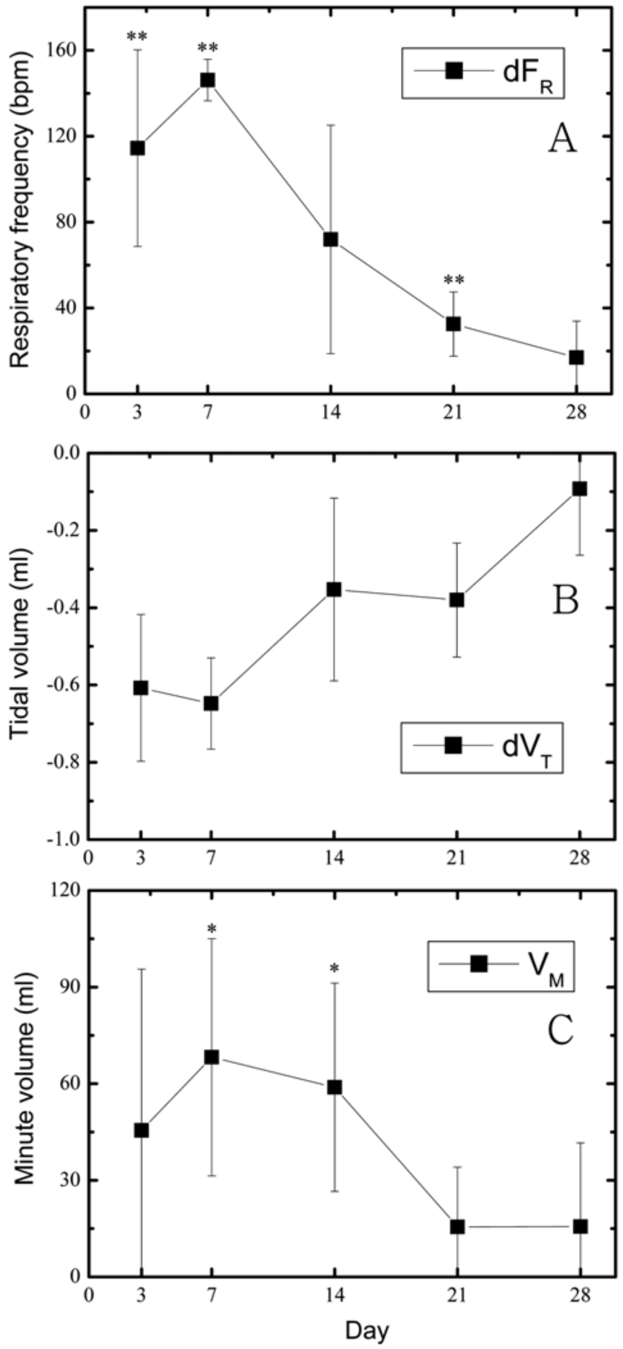

**Fig. 2.** Pulmonary function test during experimental period, (A) respiratory frequency ($F_R$), (B) tidal volume ($V_T$), and (C) minute volume ($V_M$). Values are the difference between the bleomycin-treated rats and controls.

day 3, maximum at day 7, and decreased at days 14, 21, and 28, as shown in Fig. 2(A), with a similar change in total cell count and LDH level. The tidal volume change ($dV_T$) was significantly low at days 3, 7, and 21, as seen in Fig. 2(B), and gradually recovered to the value of control group over 28 days. The difference in

**Table 2.** Incidence and severity of microscopic findings in the lung

| | Day 3 | Day 7 | Day 14 | Day 21 | Day 28 |
|---|---|---|---|---|---|
| ***Inflammatory cell infiltration*** | | | | | |
| Saline | 0 | 0 | 0 | 0 | 0 |
| Bleomycin | 2.8 ± 0.44** | 4.8 ± 0.44** | 1.6 ± 0.89* | 1.2 ± 0.44* | 1.4 ± 0.89* |
| ***Pulmonary fibrosis*** | | | | | |
| Saline | 0 | 0 | 0 | 0 | 0 |
| Bleomycin | 0 | 0 | 2.4 ± 0.89** | 2.2 ± 0.83** | 2.2 ± 0.83** |

Data presented as mean ± S.D of groups.
*$p < 0.05$ vs. saline control group.
**$p < 0.01$ vs. saline control group.

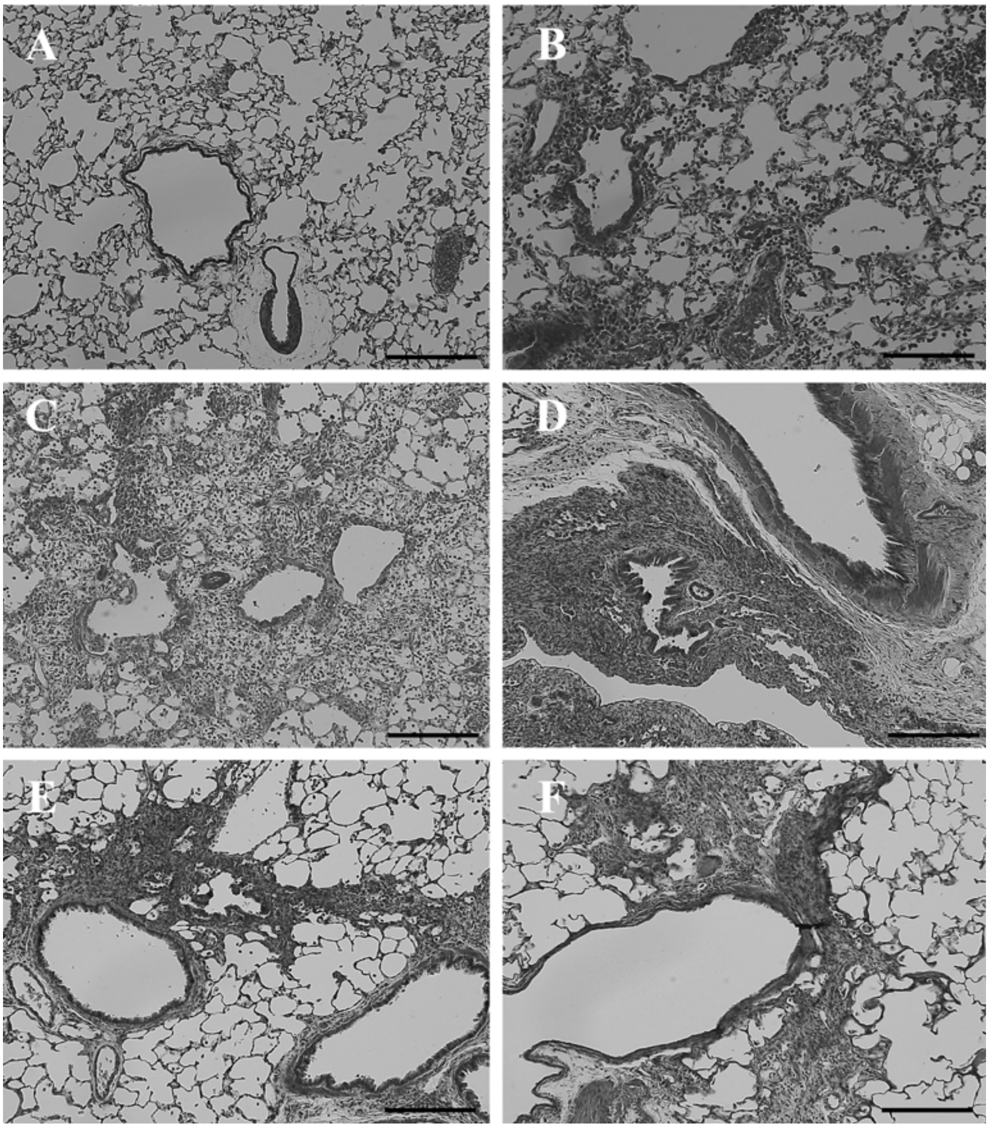

**Fig. 3.** Representative lung photomicrograph of bleomycin-instilled rat, (A) Control, (B) 3 day, (C) 7 day, (D) 14 day, (E) 21 day, and (F) 28 day. The scale bar is 200 μm.

minute volume ($dV_M$) was significantly ($p < 0.05$) high at days 7 and 14, as shown in Fig. 2(C), and reached a plateau after day 21. This is very similar to the change in total cell, LDH level, and $dF_R$.

***Histopathological findings.*** After intratracheal instillation of BLM, the histopathological changes in the lung were evaluated on days 3, 7, 14, 21, and 28. The time course of the histopathological changes in the lungs of the BLM-treated animals is illustrated in Table 2. To confirm the histopathology of BLM-induced lung inflammation and fibrosis, the overall grade of lung inflammatory and fibrotic changes was scored on days 3, 7, 14, 21, and 28. The inflammatory scores of the lung sections from BLM-treated rats peaked on day 7 and decreased thereafter. In contrast, the fibrosis observed in the BLM group started on day 14 and the scores remained steady thereafter.

Representative photomicrographs are presented in Fig. 3. Histopathological assessment showed that there was significant infiltration of inflammatory cells, mainly neutrophils, in the alveolar wall and spaces at 3 days post-ITI of BLM, as shown in Fig. 3(B). These changes were absent in lungs from saline-ITI rats as observed in Fig. 3(A), and thus are considered specific early inflammatory changes in response to BLM. On day 7, large infiltration of neutrophils, alveolar macrophages, and lymphocytes in the peribronchiolar, perivascular, and alveolar spaces, together with thickening of the alveolar interstitium were seen [Fig. 3(C)]. These changes observed at day 7 were more severe than those observed at day 3 and were most prominent. In some animals, mild infiltration of inflammatory cells was observed in respiratory bronchioles with hyperplasia of fibroblasts. At day 14, the number of inflammatory cells was low while the number of fibroblasts was high, and a mild collagenous deposit with infiltration of neutrophils and monocytes was seen. Also, interstitial thickening was observed in the alveolar walls as shown in Fig. 3(D). The severity of these changes varied slightly from rat to rat, ranging from minimal to moderate. At days 21 and 28, mild alveolar fibrosis and minimal infiltration of inflammatory cells, mainly of neutrophils and few alveolar macrophages was observed as shown in Fig. 3(E) and (F).

## DISCUSSION

BLM is a complex of related glycopeptide antibiotics from *Streptomyces verticillus* (Moeller *et al.*, 2008) that induces DNA strand scission and cellular/tissue injury. This effect is usually restricted to the lungs and skin as a result of lower levels of BLM hydrolase in these organs (Gharaee-Kermani *et al.*, 2005). The single ITI of BLM is a widely used method for inducing experimental pulmonary fibrosis in rats (Lazenby *et al.*, 1990; Izbicki *et al.*, 2002; Gharaee-Kermani *et al.*, 2005; Moeller *et al.*, 2008) and has been applied to study the mechanism and treatment of lung fibrosis (Tzurel *et al.*, 2002; El-Medany *et al.*, 2005; Iraz *et al.*, 2006).

One of the main aims of this study was to noninvasively monitor the severity of disease in the experimental model of BLM-induced lung fibrosis using PFT. It is very important to determine the onset and severity of inflammation and fibrosis in studies of pathogenesis and therapies (Karmouty-Quintana *et al.*, 2007). The reason is that the severity of induced disease models with the same dose of BLM applied in this study (2.5 mg/kg, B.W.) may be different from the genetic specificity within a species, the experimental environments, and the skillfulness of instillation. Therefore, the noninvasive monitoring method is definitely needed for the selection of proper severity without any sacrifice of experimental animals.

Karmouty-Quintana *et al.* performed magnetic resonance imaging (MRI) to noninvasively monitor lung injury to assess structural changes following a single ITI of BLM (Karmouty-Quintana *et al.*, 2007). Inflammation and morphological changes have been effectively used as lung parenchymal signals, indicating that MRI is a good tool to evaluate the therapeutic effect in experimental models of lung fibrosis in rats.

PFT is one of the possible ways to noninvasively monitor the physiologic state of animals. Further, it is very sensitive to inflammation in lungs of rats exposed to gasoline engine exhaust, which indicates a correlation to the cellular changes in BALF (Seagrave *et al.*, 2008). The temporal influx of inflammatory cells in lungs increases the respiratory frequency ($F_R$) (Spond *et al.*, 2004).

In the present study, PFTs of tidal volume ($V_T$), respiratory frequency ($F_R$), and minute volume ($V_M$) were introduced to monitor the progress of BLM-induced lung fibrosis animal models. There is a significant correlation between the changes in BALF analyses and PFT of $F_R$, $V_T$, and $V_M$, as shown in Figs. 1 and 2. The $dF_R$ and $dV_M$, in particular, increase from day 1 to 7 and decrease after day 7 similar to the changes in total cell count and LDH level. The $dV_T$ decreases significantly in Fig. 2(B), reflecting the influx of inflammatory cells into the alveoli, and edema and hyperemia in the lung parenchyma at days 3 and 7, and slowly increases to the value of controls over 28 days with the recovery of inflammation. Decrease in $V_T$ leads to the increase in

$F_R$ as shown in Fig. 2(A) in order to maintain the oxygen supply. Furthermore, more oxygen supply is needed due to the inflammatory cell influx so that the $dF_R$ and $dV_M$ peak at day 7 as shown in Fig. 2(A) and 2(C). The recovery of inflammation makes them low at days 21 and 28. The cellular changes in BALF are consistent with the previous studies on the BLM-induced pulmonary fibrosis (Izbicki *et al.*, 2002). It shows the typical progress of BLM-induced lung injury. These results are supported by the histological characteristics of the lung injury in Fig. 3 and Table 2 (3 to 28 days post BLM). These results are similar to those of the previous studies (Izbicki *et al.*, 2002; Gharaee-Kermani and Phan, 2005) and are also consistent with the results of PFT.

Moreover, the fibrotic changes start at 14 days post-BLM instillation and the pathological appearance is sustained thereafter, based on the histological consideration. Masson's trichrome staining of lung tissue demonstrated fibrosis at 14 days after treatment.

From the above consideration of BALF analyses and histological results, inflammation is induced immediately after single ITI of BLM, and fibrosis is observed after the recovery of inflammation after day 14. The parameters of PFT increase in accordance with the onset of inflammation and decrease with its recovery. The correspondence between the PFT parameters and the other results in this study support the use of BLM instillation as a good noninvasive monitoring tool for the assessment of the progress of lung injury. Selection of appropriate subjects of lung inflammation and fibrosis without any sacrifice makes the study of the pathogenesis and the therapies more efficient and economic by overcoming the genetic specificity and the difference in skillfulness, etc. Further studies are needed for correlations between the PFT and lung disease models for noninvasive monitoring.

In summary, lung fibrosis can be found after single ITI of BLM based on the histopathological consideration. Hence, the most suitable time point for observing the effect of therapeutic agents is 14 days post BLM treatment. PFT of $F_R$, $V_T$, and $V_M$ can be a very good tool for monitoring the progress of lung injury induced by BLM treatment and can support a practical and noninvasive standard to assess if the experimental models are well developed.
